# A discrete event simulation approach for reserving capacity for emergency patients in the radiology department

**DOI:** 10.1186/s12913-018-3282-8

**Published:** 2018-06-15

**Authors:** Li Luo, Yumeng Zhang, Fang Qing, Hongwei Ding, Yingkang Shi, Huili Guo

**Affiliations:** 1 Business School, Sichuan University, Chengdu, 610064 Sichuan China; 2 West China Hospital, Sichuan University, Chengdu, Sichuan China

**Keywords:** Discrete event simulation, Emergency reservation policy, Decision making, Appointment scheduling

## Abstract

**Background:**

Many hospitals in China experience large volumes of emergency department (ED) radiology patients, thereby lengthening the wait times for non-emergency radiology patients. We examine whether an emergency reservation policy which deals with stochastic arrivals of ED patients can shorten wait times, and what effect it has on patient and hospital related metrics.

**Methods:**

In this study, operations research models are used to develop an emergency reservation policy. First, we construct a discrete event simulation (DES) model based on the process of patients served by one computed tomography (CT) scanner at West China Hospital (WCH). Next, a newsvendor model is built to compute the daily reservation quantity for emergency patients. Based on the appointment scheduling rule and daily emergency reservation policies, the effects of the proposed policy on daily examination quantity, patient wait times, and equipment utilization are explicitly modeled. Finally, we evaluate the impact of different reservation policies on these system performance measures.

**Results:**

Our analysis indicates that reserving capacity for emergency patients greatly shortens the delay for non-emergency patients with an average 43.9% reduction in total wait times. The pre-model utilization and average post-model utilization are 99.3% and 98.5%, respectively. In addition, the comparison of different reservation policies shows that there is no significant difference between any two policies in terms of patients’ wait times.

**Conclusions:**

Reserving proper capacity for emergency patients not only positively affects the patients’ delay times, but also affects various aspects of the hospital. Our goal is to design a simple and implementable emergency reservation policy. DES proves to be an effective tool for studying the effects of proposed scenarios to optimize capacity allocation in radiology management.

## Background

Medical imaging equipment, such as computed tomography (CT) scanner, constitutes a critical component of a comprehensive health care system and plays a key role in the diagnosis and treatment of disease [1]. The demand for diagnostic imaging services has increased substantially over the past decades [2]. However, high operational costs and their influence in increasing medical costs have resulted in hospitals not extending their capacity at the same rate [3]. The large gap between supply and demand is associated with lengthy wait times, hospital overcrowding, and patient dissatisfaction [4, 5]. Under pressure from rising demand and costs, hospitals must take effective measures to organize their medical services to improve the service delivery process and increase patient satisfaction [6]. Therefore, effective appointment scheduling models [7–10] are inevitably of great importance.

The motivation behind this study is the practice and problem of the radiology department of West China Hospital (WCH), one of the largest hospitals in China. The normal work hours of the radiology department are 8:00–21:00 (13 h) and the utilization of the equipment is almost 100%. Generally, the medical diagnostic facilities are accessed by three types of patients. Emergency patients arrive randomly with a higher priority. Both inpatients and outpatients are required to make appointments in advance [1, 11]. The daily challenge is to allocate resources among different types of patients. The problem faced by the radiology department is that there is no scientific method to determine how much capacity should be reserved for emergency patients. Through investigation and survey, we find that there is a large imbalance between appointments and service capacity, and the average wait times of outpatients and inpatients are 163.2 min and 144.7 min, respectively. A poor reservation process and poor scheduling lead to operational inefficiency and patient dissatisfaction [12]. In contrast, the overall goal of a well-designed appointment system is to achieve a balance among the competing and conflicting goals of minimizing the patients’ wait times and the doctor’s idle time and overtime [13]. Thus, the above analysis highlights the need for operations research models that provide a scientific emergency reservation policy for hospital managers.

In this study, we use simulation and optimization techniques to solve the described management problems in a complex health care system. The analysis is conducted using primary data from WCH and the following research questions are addressed: (1) How much daily and hourly capacity should be reserved for emergency patients? (2) Do different reservation policies have an influence on non-emergency patients’ wait times? (3) Are there any significant differences between different reservation policies? Through combined optimization with a validated discrete event simulation (DES) model, we evaluate the impact of different emergency reservation policies on a variety of system outcomes. On the basis of this work, practical guidance can be provided on resource allocation and appointment scheduling decisions.

## Methods

### Simulation model

We construct the DES model based on the process of patients served by one CT scanner at WCH. An overview follows of the process of patient flow, performance measures, model assumption, data collection and analysis, and model validation. Finally, we determine the best scheduling rules and provide sensitivity analysis.

**Fig. 1:**
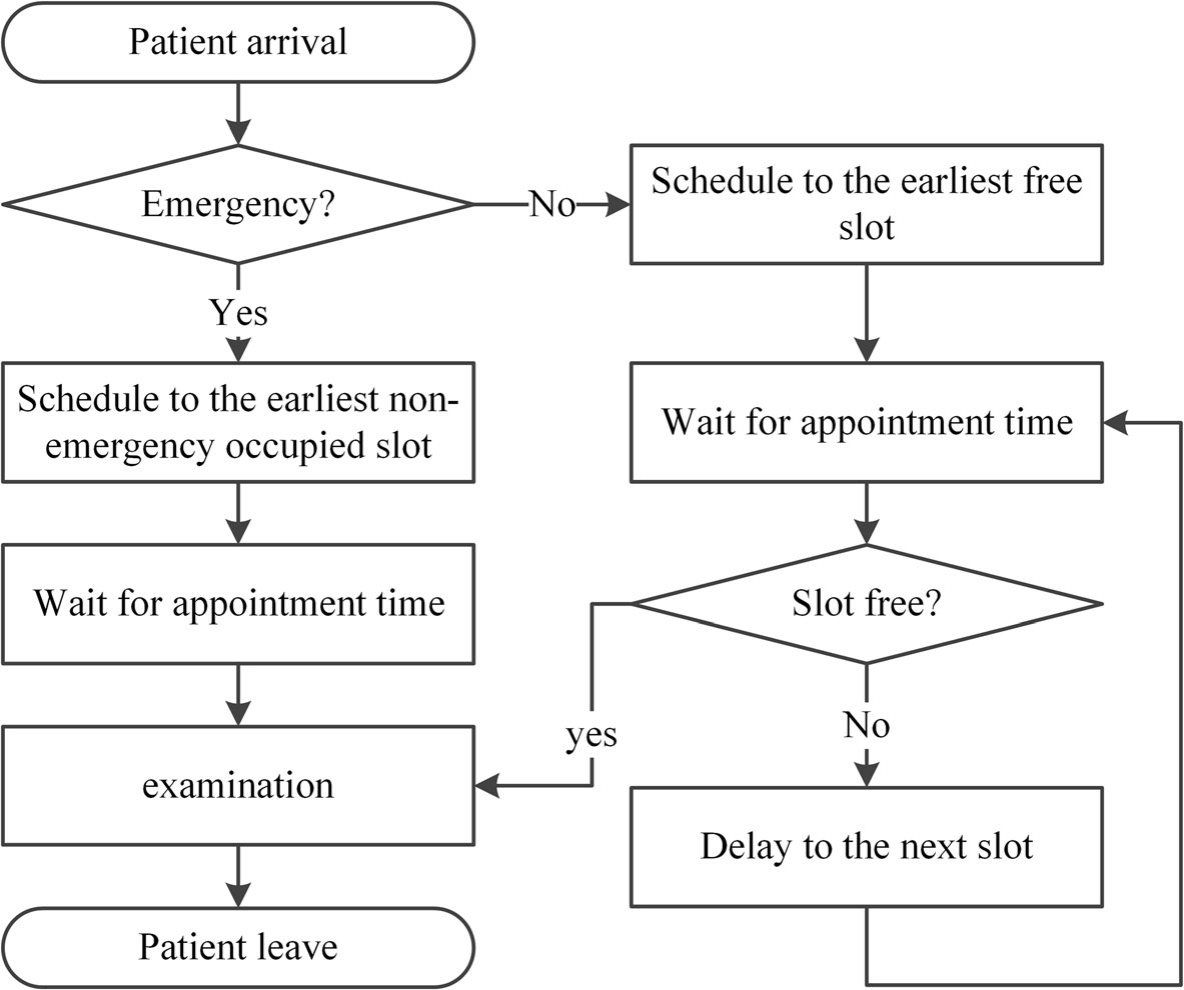
The process for patients to accept CT examination

**Fig. 2:**
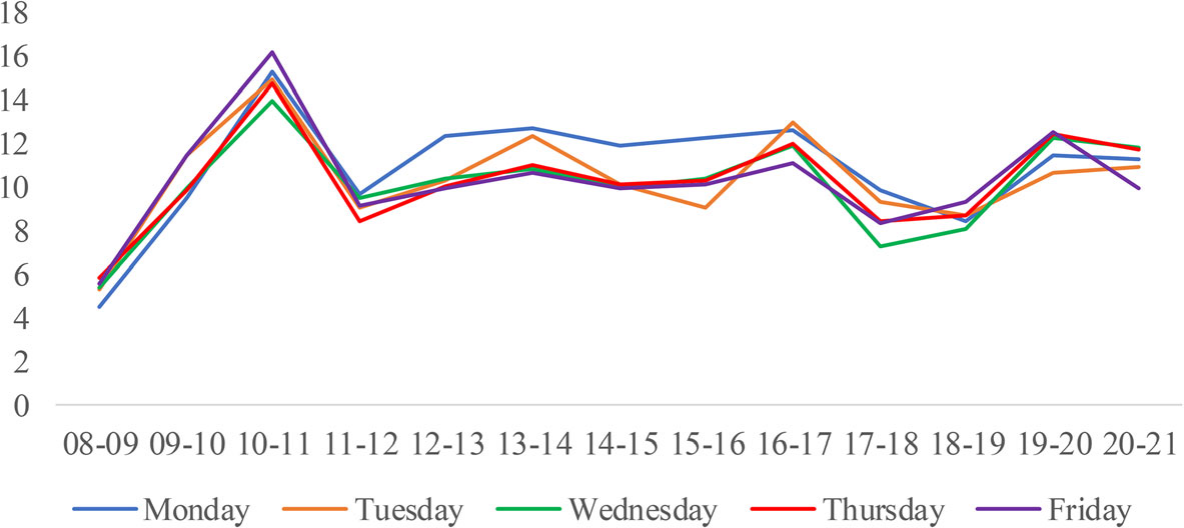
The arrival pattern of emergency patients during workday

**Fig. 3:**
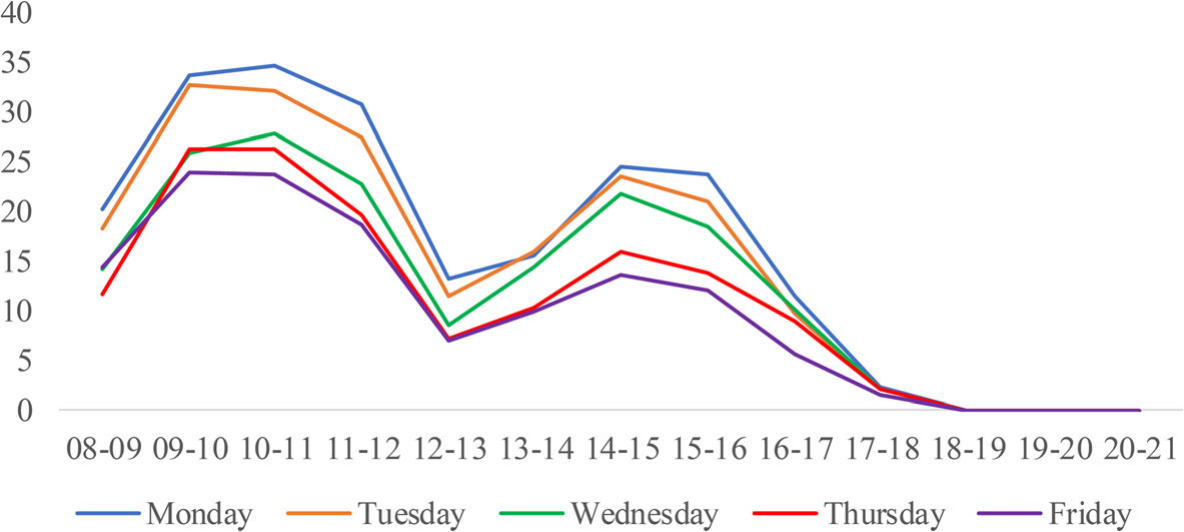
The arrival pattern of outpatients during workday

**Fig. 4:**
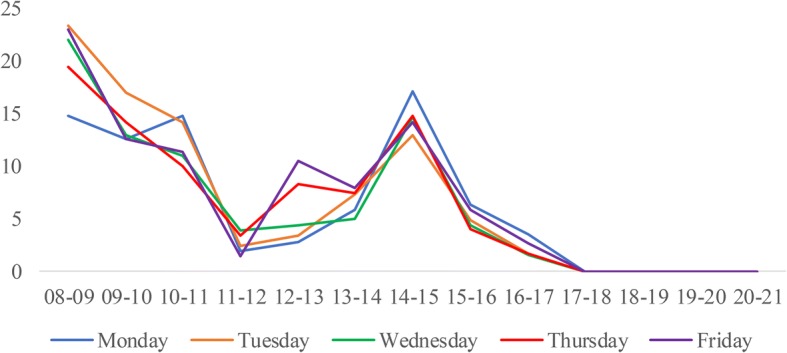
The arrival pattern of inpatients during workday

1. **Patient flow process**. Figure 1 shows the process of patients served by one CT scanner, which provides routine scans to three types of patients. If new emergency patients arrive, they are scheduled in the earliest non-emergency occupied slots and must wait until all emergency patients before them are served. Otherwise, they are booked and scheduled in the earliest free slots. If the slot is occupied by an emergency patient, they must wait until there are slots available.
2. **Performance measures.** The performance measures include daily examination quantity, patient wait time, and equipment utilization. All of these measures are considered because of their impacts on patient satisfaction and quality of care. Daily examination quantity refers to the service capability of the CT scanner during normal working hours. Patient wait time refers to the time between the appointment beginning and examination taking place. This measure is directly associated with patients’ satisfaction. Equipment utilization refers to the ratio of the average time the CT scanner worked and the total operational time (from 8:00 to 21:00).
3. **Model assumptions.** To simulate the scanning process, the following assumptions are made:

- Service priority. Emergency patients have a higher priority, and both outpatients and inpatients share the same lower priority;
- Service discipline. Scheduled patients are examined on a first-come-first-serve (FCFS) basis;
- Service timeframe. The normal duration of operations is from 8:00 to 21:00 daily;
- Equipment practice. The equipment will not break down during working hours;
- Patient behavior. All scheduled patients arrive on time.

(4) **Data collection and analysis.** The data obtained for the simulation model are from the Radiology Information System (RIS) of WCH. We collected over 90,000 examination records of the CT scanner from September 1st, 2011, to July 31st, 2012. The arrival time is defined as the first time a patient arriving at the service station requests a medical examination. After a comprehensive analysis of patient arrival times, we identify the day-of-week pattern and time-of-day effect across patient types, as shown in Fig. 2, Fig. 3, and Fig. 4. Then, the demand distribution of different types of patients is fitted, analyzed, and tested. The analysis is run on SPSS21.0, and the results show that the demand of each patient type in almost every period (one hour) fits the Poisson distribution. Without loss of generality, we use Wednesday as an example to account for the day-of-week effect. A distinguished feature of our model is that we allow the number of regular appointments that can be scheduled on each day to be dynamic.
  As the RIS does not record the exact examination time, we collected data over a one-week period from July 23rd, 2012, to July 29th, 2012, by tracing a combination of observers and handwritten scheduling documents containing 790 records. Table 1 gives the examination time frequency analysis of each patient type. Both the patient’s demand distribution and examination times form the input to the simulation model.

(5) **Model validation.** A single server system is designed for our simulation model, which is built using SIMIO. To validate our model, the simulation is executed 1000 times. Table 2 lists the outputs of the baseline model. At a confidence level of 95%, the simulation model is consistent with the actual results.

**Table 1:** The examination times of three types of patients

Examination time (min)	Emergency patients	Outpatients	Inpatients
	frequency	frequency	frequency
1.00	7.6%	12.4%	12.9%
2.00	41.0%	59.3%	56.1%
3.00	37.2%	21.7%	27.7%
4.00	14.2%	6.6%	3.3%

**Table 2:** Outputs of the baseline model

Performance measures	Outputs		
	Historical data	Baseline model	95% confidence interval of mean value
Examination quantity	327.0	327.4	(326.5, 328.9)
Wait time	83.4	83.8	(82.4, 83.9)
Equipment utilization	≥99%	99.5%	(98.9%, 99.8%)

### Optimization model

Based on the scheduling rule determined by the above simulation model, the classical newsvendor model is used to determine the daily reservation policy for emergency patients.

1. **Daily reservation policy.** A classical newsvendor model determines the daily reservation policy for emergency patients (every Wednesday). Suppose *E* is the decision variable. When the actual emergency demand is less than *E*, an idle time cost is incurred. Otherwise, the emergency patients occupy the non-emergency patients’ slots, which leads to an overtime cost. Our goal is to determine the optimal value of *E* that minimizes the sum of idle and overtime costs.
  Hospital managers generally prefer to work overtime rather than keep equipment idle, though they must pay additional fees for working overtime. Without a loss of generality, let the cost ratio of the idle cost to overtime cost be 2, and the maximum overtime slots be 30 slots. The newsvendor model is formulated by

1 $$ {\displaystyle \begin{array}{l}\kern2.25em \mathit{\operatorname{Min}}\kern0.5em TC=2E(ES)+E(OT)\\ {}\kern2.25em Subject\ to\kern0.75em E(OT)\le 30\\ {}\kern2.5em E(ES)=\sum \limits_{k=0}^Ek\cdot P\left({D}_E=E-k\right)\\ {}\kern6em =\sum \limits_{k=0}^Ek\cdot \frac{{\left({\lambda}_E\right)}^{E-k}{e}^{-{\lambda}_E}}{\left(E-k\right)!}\\ {}\kern2.5em E(OT)=\sum \limits_{k=0}^{\infty }k\cdot P\left({D}_E=E+k\right)\\ {}\kern6em =\sum \limits_{k=0}^{\infty }k\cdot \frac{{\left({\lambda}_E\right)}^{E+k}{e}^{-{\lambda}_E}}{\left(E+k\right)!}\end{array}} $$

where *D*_*E*_ is a random variable representing the emergency arrivals with Poisson distribution *F*_*E*_, and *λ*_*E*_ is the corresponding parameter (*λ*_*E*_ = 131). The variables *TC*, *ES*, and *OT* are the expectations of the total cost, idle cost, and overtime cost, respectively. Finally, we solve Eq. (1) using MATLAB. The total cost reaches the minimum when 135 slots are reserved for emergency patients.

(2) **Hourly reservation policy.** As the arrival pattern displays the time-of-day effect, the arrivals of emergency patients are modeled as non-stationary Poisson processes. The hourly reservation policy is based on the statistical results of each hour, as listed in Table 3.

**Table 3:** Hourly reservation policy for emergency patients

8–9	9–10	10–11	11–12	12–13	13–14	14–15	15–16	16–17	17–18	18–19	19–20	20–21
6	12	15	10	10	11	10	10	12	10	8	10	11

We then propose four reservation policies according to the position of reservations in each hour: the initial stage of an hour, end stage of an hour, intermediate stage of an hour, and average distribution in an hour, as given in Table 4. In this simulation model, patient schedules are generated by varying the number of emergency patients in each hour with four reservation policies.

**Table 4:** The proposed reservation policies for emergency patients

Cases	Schedule rule	Reservation rule for emergency patients	
		Yes/no	Position
Base Case	(2, 1, 0)	No	–
Case 1	(2, 1, 0)	Yes	the initial stage of an hour
Case 2	(2, 1, 0)	Yes	the end stage of an hour
Case 3	(2, 1, 0)	Yes	intermediate stage of an hour
Case 4	(2, 1, 0)	Yes	average distributed in an hour

## Results

### Scheduling rules

As the average examination time varies from 1 to 2.5 min, five scheduling rules (*a*, *n*, *n*_1_) are used to simulate and compare with the actual case, as given in Table 5. The first element *a* in the scheduling rule refers to the appointment time interval, the second element *n* refers to the number of patients making appointments during the appointment time interval *a*, and the third element *n*_1_ refers to the number of patients making appointments at the beginning. Simulation results show that scheduling one patient every 2 min achieves a better balance among three performance measures, as given in Table 6. Thus, the rule (2,1,0) is the basic appointment scheduling rule of this study.

**Table 5:** The proposed appointment scheduling rules

(*a*, *n*, *n*_1_)	Appointment scheduling rules
(1, 1, 0)	Scheduling 1 patient each min
(3, 2, 0)	Scheduling 2 patients every 3 min
(2, 1, 0)	Scheduling one patient every 2 min
(12, 5, 0)	Scheduling 5 patients every 12 min
(5, 2, 0)	Scheduling 2 patients every 5 min

**Table 6:** Outputs of the five appointment scheduling rules

Performance measures		Rule 0	Rule 1 (1,1,0)	Rule 2 (3,2,0)	Rule 3 (2,1,0)	Rule 4 (12,5,0)	Rule 5 (5,2,0)
Examination quantity	Total	327.0	327.5	326.4	326.9	317.5	307.8
	Emergency patients	132.6	133.4	133.8	132.6	131.4	132.2
	Outpatients	129.3	127.2	125.4	127.5	123.5	116.2
	Inpatients	65.1	66.9	67.1	66.9	62.6	59.4
Wait time (min)	Total	83.4	128.5	108.6	53.9	6.6	2.5
	Emergency patients	3.7	2.3	2.4	1.8	2.3	1.2
	Outpatients	163.2	233.4	198.0	96.6	10.8	3.6
	Inpatients	144.7	184.8	160.8	78.0	9.6	3.0
Equipment utilization		≥99%	99.5%	99.2%	99.3%	96.0%	93.6%

### Sensitivity analysis

To analyze the influence of two factors (patient arrival rate and service time) on performance measures, we conduct sensitivity analysis. The goal is to identify the value of the two factors to help derive effective radiology appointment scheduling rules. As emergency patients have a higher priority, we consider the changes in emergency arrival rates and the mean service times of three types of patients.

**Fig. 5:**
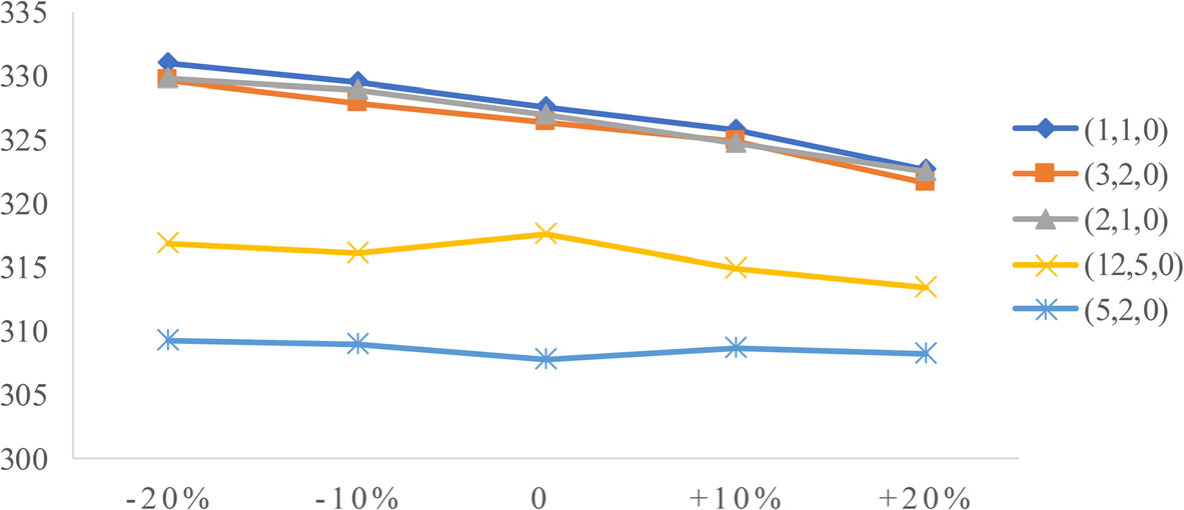
Impact of different emergency arrival rate on examination quantity

**Fig. 6:**
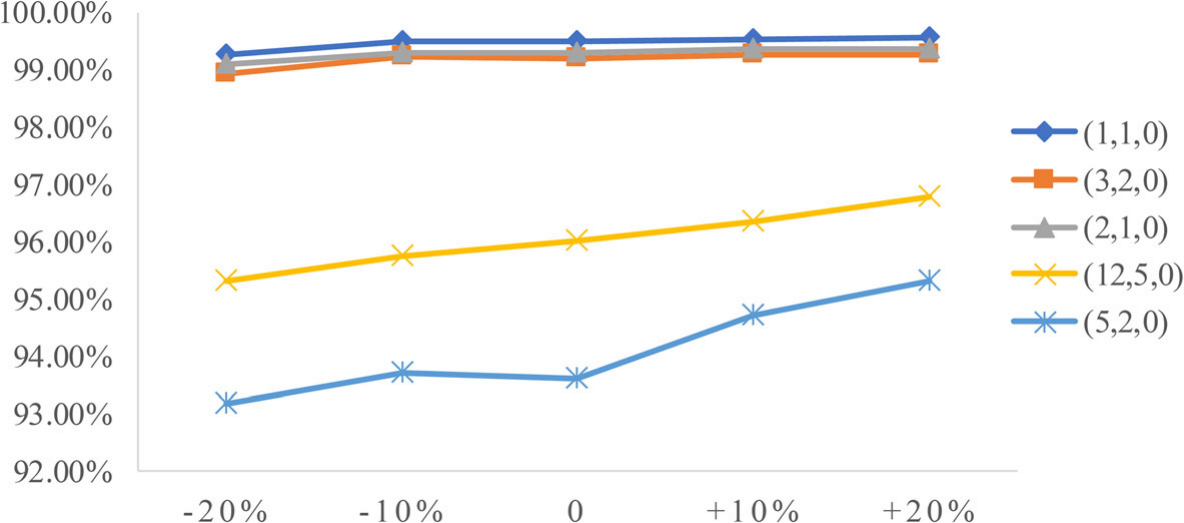
Impact of different emergency arrival rate on equipment utilization

**Fig. 7:**
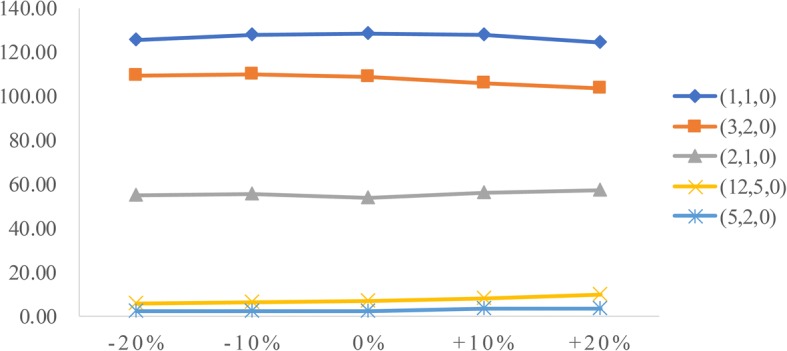
Impact of different emergency arrival rate on total wait times

**Fig. 8:**
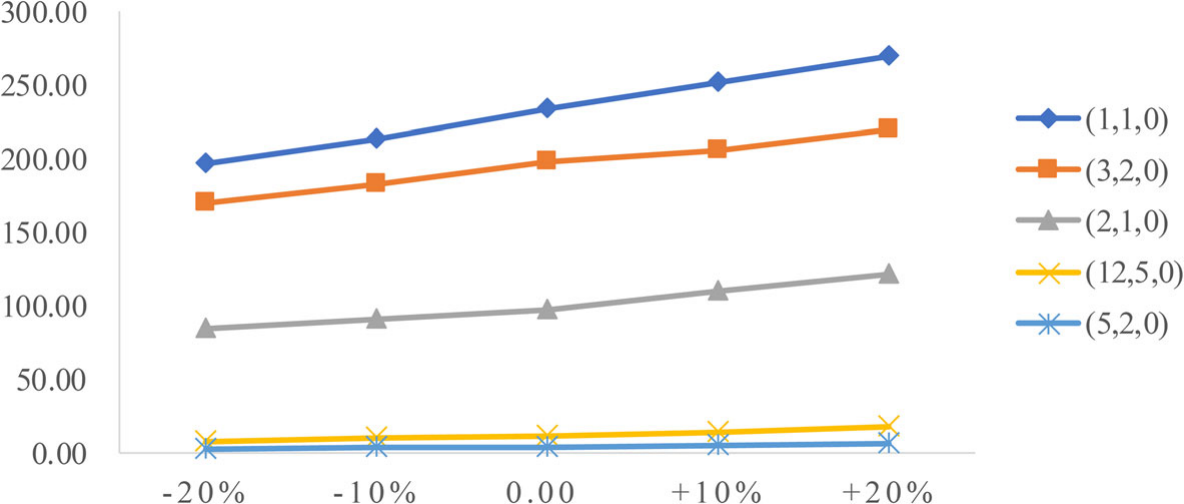
Impact of different emergency arrival rate on outpatients’ wait times

**Fig. 9:**
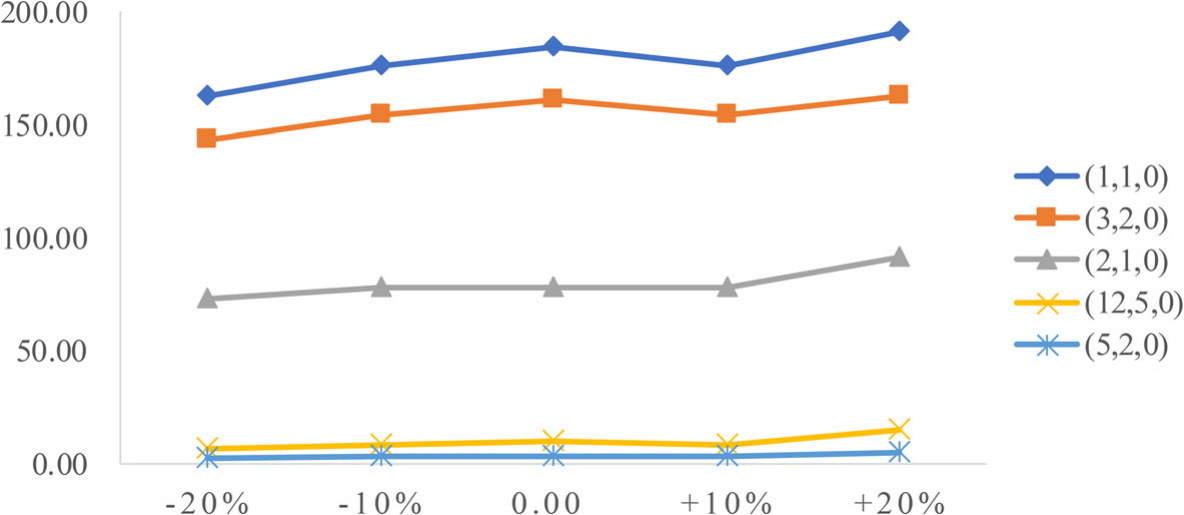
Impact of different emergency arrival rate on inpatients’ wait times

**Fig. 10:**
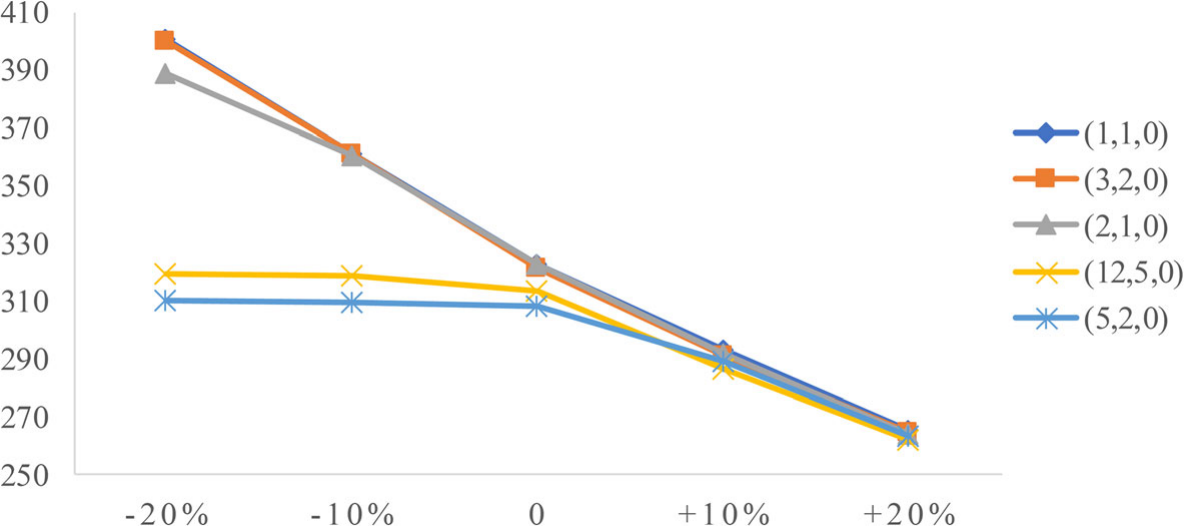
Impact of different mean service times on examination quantity

**Fig. 11:**
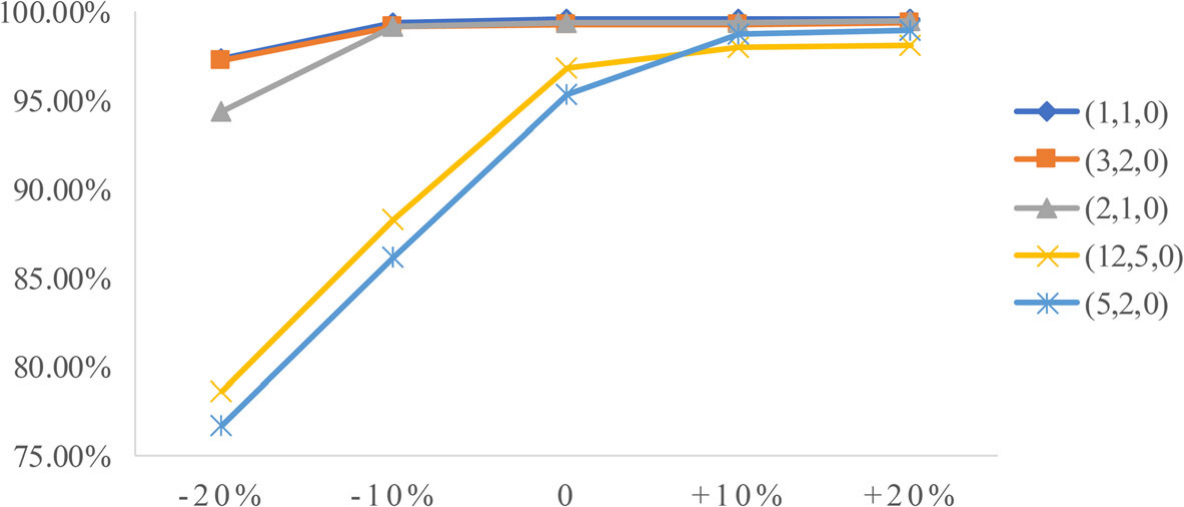
Impact of different mean service times on equipment utilization

**Fig. 12:**
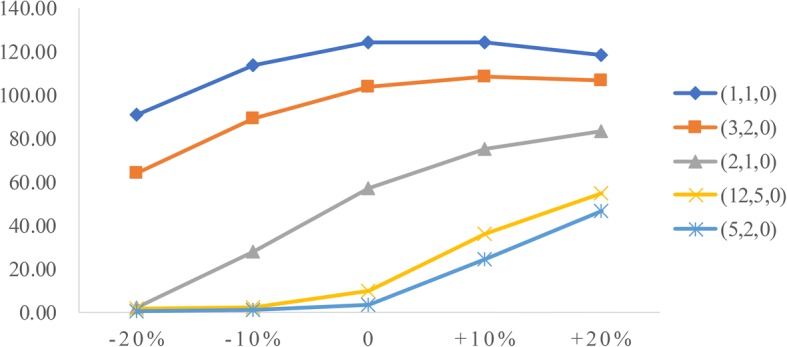
Impact of different mean service times on total wait times

1. **Patient arrival rate.** Let the emergency arrival rates fluctuate over a range of plus or minus 20%. The results are displayed in Figs. 5, 6, 7, 8, 9. As the emergency arrival rate increases, the examination quantity decreases from 329.8 to 322.5, the equipment utilization increases from 99.10% to 99.37%, and the total wait times increase from 54.96 to 57.04 min according to the rule (2,1,0). The wait times of outpatients and inpatients increased rapidly, indicating that the emergency arrival rate has a significant effect on performance measures.
2. **Service time.** Similarly, let the mean service times fluctuate over a range of plus or minus 20%. The results are shown in Figs. 10, 11, 12. As the mean service time increases, the examination quantity decreases from 388.3 to 264.2, the equipment utilization increases from 94.39% to 99.46%, and the total wait times increase from 2.04 to 83.26 min according to the rule (2,1,0).

The analysis highlights that both factors have great effects on the system performance. Because the mean service time is mainly determined by technicians’ skill and patients’ degree of compliance, we only consider the issue of designing an emergency reservation policy to minimize the non-emergency patients’ wait times.

### Impact of reservation policies on system outcome

By establishing simulation models with varied emergency reservation policies, we obtain the results summarized in Table 7. The output of the base case refers to the system output according to the rule (2,1,0), where emergency reservation is not considered. We compare the base case and four reservation policies to show the performance improvements facilitated by reserving capacity for emergency patients.

1. **Examination quantity.** There is a slight decrease in examination quantity compared to the base case. The percentages of reduction in examination quantity are 1.7%, 0.7%, 0.9%, and 0.9%, respectively.
2. **Patient wait time.** Surprisingly, the wait times of different types of patients decrease significantly. The percentages of reduction in total wait times from case 1 to case 4 are 45.6%, 43.7%, 42.3%, and 44.1%, respectively. For outpatients and inpatients, the reduction in wait times exhibits the same trends.
3. **Equipment utilization.** The average equipment utilization of all cases is 98.5%, indicating there is a slight reduction compared to the base case.

**Table 7:** Outputs of different reservation policies for emergency patients

Performance measures		Base case	Case 1	Case 2	Case 3	Case 4
Examination quantity	Total	326.9	321.3	324.6	324.0	324.0
	Emergency patients	132.6	134.1	133.2	132.7	132.0
	Outpatients	127.4	122.3	126.2	125.4	125.0
	Inpatients	66.8	64.9	65.1	65.9	67.0
Wait time (min)	Total	53.9	29.3	30.4	31.1	30.1
	Emergency patients	1.8	2.0	1.9	2.0	1.9
	Outpatients	96.6	53.6	54.7	56.6	54.8
	Inpatients	78.0	43.0	44.5	45.8	44.2
Equipment utilization		99.3%	97.9%	98.8%	98.5%	98.7%

Furthermore, one-way analysis of variance and multiple comparisons are applied to explore whether there are significant differences among different reservation policies. As indicated in Table 8, there are significant differences between case 1 and cases 2–4 in terms of examination quantity and equipment utilization. In contrast, there is no significant difference between any two cases from the perspective of wait times.

**Table 8:** Performance comparisons of different reservation policies

Examination quantity		Equipment utilization		Total wait time		Wait times of outpatients		Wait times of inpatients	
Case 2	324.6	Case 2	98.8%	Case 1	29.3	Case 1	53.6	Case 1	43.0
Case 3	324	Case 4	98.7%	Case 4	30.1	Case 2	54.7	Case 4	44.2
Case 4		Case 3	98.5%	Case 2	30.4	Case 4	54.8	Case 2	44.5
Case 1	321.3^**^	Case 1	97.9%^**^	Case 3	31.1	Case 3	56.6	Case 3	45.8

**p ≤ 0.05

## Discussion

When facing uncertain patient volume, effective schedule management is key to minimizing patient wait times, improving equipment utilization, and reducing the overall system cost. Radiology department scheduling is more challenging than outpatient [7] or operating room scheduling [8, 9, 14, 15], as it involves both appointed and emergency patients [16]. This study focuses on the allocation of medical capacity in the presence of multiple patient classes. Our goal is to design a simple and implementable emergency reservation policy. By combining optimization with DES, we evaluate the impact of different reservation policies on patient and hospital related metrics. Based on our work, practical guidance for appointment scheduling and emergency reservation can be provided.

Results demonstrate that although there is a slight decrease in examination quantity and equipment utilization, reservations for emergency patients greatly shorten the delay for non-emergency patients, with a reduction of over 40% in wait times. Furthermore, the comparison of different reservation policies indicates that they are relatively robust in terms of patients’ wait times. Our results differ from Erdogan et al. [17], who determined that reserving the capacity for urgent patients at the beginning of the session is favorable. Their results were obtained under the condition that the wait costs of urgent patients are high, whereas our study highlights the value of idle time.

The issue of redesigning the appointment scheduling rule and reserving capacity for emergency patients has attracted much attention in recent years. Green et al. [1] investigated a related problem of appointment scheduling and resource allocation for one medical diagnostic facility. They formulated the problem of managing patient demand for diagnostic service as a finite-horizon dynamic program, and identified properties of the optimal policies. Kolisch and Sickinger [18] extended [1] to the multiple facility setting, where they showed that the optimal policy is not simple but exhibits desirable monotonicity properties. Due to the computational complexity, it’s not easy for hospitals to implement the state-dependent optimal policy in practice.

As a result, simulation is an effective tool for allocating scarce resources to improve patient flow, while minimizing health care delivery costs and increasing patient satisfaction. A comprehensive literature review on the use of simulation in health care can be found in [19], where the application of DES modeling to health care and system clinics, such as hospitals, outpatient clinics, emergency departments and pharmacies is summarized. Bhattacharjee and Ray [20] discussed the reasons for selecting DES in the context of health care decision-making processes. Monks et al. [21] developed a DES model for capacity planning in acute and community stroke services. Lebcir et al. [22] adopted a DES model to evaluate the use of community services in the treatment of patients with Parkinson’s disease in the United Kingdom. Vermeulen et al. [23] presented an adaptive approach to automatic optimization of resource calendars, and developed a simulation model to determine optimal resource opening hours in a larger time frame where the allocation of capacity to different patient groups is flexible and adaptive to current and expected future situations. Antognini et al. [24] proposed a Monte Carlo simulation model to guide decisions on how to balance resources for elective and non-elective surgical procedures. This study also provided a simulation approach to radiology patient scheduling, and our results showed that DES is a potent tool for process improvement in health care systems.

The limitations of this study can be summarized as follows. First, we do not consider patients’ behaviors such as no-shows and unpunctuality [25, 26], the inclusion of which would make our simulation model much more practical. Second, our DES model is constructed based on the patient process of one CT scanner, we may extend our model to multiple facilities in the future.

## Conclusions

The management of medical imaging resources is challenging because of uncertain demand. To minimize patient wait times in the face of this uncertainty, we proposed a DES approach to radiology patient appointment scheduling. Our results indicated that reserving proper capacity for emergency patients reduces delays and improves operational metrics. DES is an effective tool for studying the effects of proposed scenarios on radiology capacity allocation, and can help to optimize hospital resource utilization.

## Abbreviations

CT: Computed tomography
DES: Discrete event simulation
ED: Emergency department
FCFS: First-come-first-serve
RIS: Radiology Information System
WCH: West China Hospital
